# Using Wood Rot Phenotypes to Illuminate the “Gray” Among Decomposer Fungi

**DOI:** 10.3389/fmicb.2020.01288

**Published:** 2020-06-12

**Authors:** Jonathan S. Schilling, Justin T. Kaffenberger, Benjamin W. Held, Rodrigo Ortiz, Robert A. Blanchette

**Affiliations:** ^1^Department of Plant & Microbial Biology, University of Minnesota, Saint Paul, MN, United States; ^2^Department of Bioproducts & Biosystems Engineering, University of Minnesota, Saint Paul, MN, United States; ^3^Department of Plant Pathology, University of Minnesota, Saint Paul, MN, United States; ^4^Escuela de Construcción Civil, Facultad de Ingeniería, Universidad de Valparaíso, Valparaíso, Chile

**Keywords:** gray rot, brown rot, white rot, soft rot, decomposer, peroxidase, CAZY, decay

## Abstract

Wood-decomposing fungi use distinct strategies to deconstruct wood that can significantly vary carbon release rates and fates. White and brown rot-type fungi attack lignin as a prerequisite to access carbohydrates (white rot) or selectively remove carbohydrates (brown rot). Soft rot fungi use less well-studied mechanisms to deconstruct wood (e.g., cavitation and erosion). These fungi often co-exist in nature, creating a balance in carbon turnover that could presumably “tip” in a changing climate. There is no simple genetic marker, however, to distinguish fungi by rot types, and traditional black and white distinctions (brown and white, in this case) cannot explain a spectrum of “gray” carbon loss possibilities. In this study, we tested 39 wood-degrading fungal strains along this spectrum of rot types. We tracked wood mass loss and chemical changes in aspen blocks in early- to mid-decay stages, including three signatures of fungal nutritional mode measured from wood rather than from fungus: dilute alkali solubility, water-soluble monosaccharides, and lignin loss (%) relative to density loss (%) (L/D). Results were then plotted relative to rot types and correlated with gene counts, combining new data with past results in some cases. Results yielded a novel distinction in soluble monosaccharide patterns for brown rot fungi, and reliable distinctions between white and brown rot fungi, although soft rot fungi were not as clearly distinguished as suggested in past studies. Gene contents (carbohydrate-active enzymes and peroxidases) also clearly distinguished brown and white rot fungi, but did not offer reliable correlation with lignin vs. carbohydrate selectivity. These results support the use of wood residue chemistry to link fungal genes (with known or unknown function) with emergent patterns of decomposition. Wood signatures, particularly L/D, not only confirm the rot type of dominant fungi, but they offer a more nuanced, continuous variable to which we can correlate genomic, transcriptomic, and secretomic evidence rather than limit it to functional categories as distinct “bins.”

## Introduction

Fungi are Earth’s dominant forest decomposers, and they release carbon from our largest pool of aboveground biomass – wood. Fungal wood decay mechanisms, given their variable capacities to unlock carbohydrates embedded within lignin, dictate the flow of recycled carbon in forests and promise gene targets for industries. Harnessing fungal DNA information to predict decay rates and wood chemical changes is thus a critical need. Reliable predictions of wood decay using DNA-based information, however, remains both a priority (Keenan et al., 2013) and a challenge (e.g., Song et al., 2017 vs. Cline et al., 2018).

Connecting fungal genomes to their emergent wood decomposition functions is an inexact science, largely due to biological variability. Fungal nutritional strategies for deconstructing wood are not functionally redundant – they vary in ways that can have major implications in ecosystems and on greenhouse gas emissions. White rot-type fungi target lignin with enzymes such as peroxidases (PODs) to unsheathe carbohydrates and gain access for carbohydrate-active enzymes (CAZYs) such as glycosyl hydrolases (GHs) targeting hemicellulose and cellulose. Brown rot fungi instead use reactive oxygen species (ROS) mechanisms to loosen wood cell wall components before selectively extracting carbohydrates, using a contracted set of GHs expressed at higher levels (Zhang et al., 2019). A third group, soft rot fungi, use less well-studied cavitation or erosion mechanisms to mine carbohydrates from the lignocellulose matrix (Eriksson et al., 1990; Daniel and Nilsson, 1998; Blanchette et al., 2010). These decay types, particularly the lignin in residues, will influence the succession of wood decay and soil community assembly and function (Zeikus, 1981; Cornwell et al., 2008; Talbot et al., 2015). These various fungal nutritional modes also alter wood solubility (Cowling, 1961; Worrall et al., 1997; Schilling et al., 2015), lignin methylation (Filley et al., 2002), and strength (Brischke et al., 2008) in unique ways. These unique pathways steer carbon toward different fates that could significantly alter CO_2_ emissions from a given forest.

Within and among these distinct rot types, there is a great deal of gray that confounds the binary brown vs. white approach to rot type classification. One well-known example is among white rot fungi, where some species exhibit a selective lignin degradation mode and others a simultaneous degradation of all wood structural bio-polymers (Blanchette, 1984, 1991). Some individual white rot species are capable of exhibiting either selective or simultaneous decay, depending on substrate and environmental conditions (Otjen et al., 1987). This kind of variability and plasticity can have great consequence on the amounts and rates of carbon released from wood. We have also learned that wood-degrading fungi greatly differ in the type and number of enzymes at their disposal for lignocellulosic degradation (Eastwood et al., 2011; Floudas et al., 2012; Riley et al., 2014), in some cases without clear genomic explanations for observed lignocellulolytic capacity. Brown rot fungi do not appear to show this degree of mechanistic diversity (Kaffenberger and Schilling, 2014), but in many ways resemble soft rot fungi in terms of carbohydrate selective mechanisms and cubical checking during wood decay (Eriksson et al., 1990; Blanchette et al., 2004; Abdel-Azeem et al., 2019).

From the inadequacy of these rot type categories (bins), the concept of “gray rot” has emerged, particularly in reference to the inadequacy of the brown vs. white rot paradigm (Riley et al., 2014). However, the meaning of the word “gray” is regularly used to denote two different things: (1) inadequate gene-to-function linkages (for example, fungus lacks POD genes but degrades lignin) and (2) a gradient of carbohydrate selectivity rather than a binary distinction (degrades lignin selectively vs. degrades lignin simultaneously with carbohydrates). The former represents an unknown – the latter represents a gradient. In the case of the latter carbohydrate selectivity gradient, a significant body of research developed in a pre-molecular era exists to demonstrate this phenotypic variability. This has not been well-linked to the former, the inadequate gene-to-functional linkages, despite its potential to correlate with genomic information and its direct link to carbon utilization.

In this study, we made phenotype rather than genotype a primary focus for our fungal wood decay trials, with the intent to establish several things we believe are useful for genomics research. First, we wanted to expand the number of isolates used in Worrall et al. (1997) and in Schilling et al. (2015) to confirm a broad distinction in carbohydrate selectivity among brown and white rot fungi. Second, we wanted to test several other wood residue qualities, specifically alkali solubility and free sugar content, that might also distinguish rot type, given the lack of reliable genetic information to do so. Third, we wanted to assess the potential for relative lignin selectivity, a continuous variable, to delineate the “gray” spectrum of rot types and offer an independent variable to correlate with gene contents. Our results demonstrate reliable distinctions between white and brown rots with all three variables (solubility, sugars, and lignin selectivity), but a need to combine tests is necessary if soft rot fungi are evaluated. In terms of gene content patterns, however, there was poor correlation between lignolytic gene content and lignolysis by white rot fungi. These results offer a useful tool that is measurable as a compiled history of rot types in the wood residues, and it can help better link fungal genes to the phenotypic traits related to functions of interest.

## Materials and Methods

### Fungal Cultures

Many fungi used in this study were field isolates collected across North and South America by the authors, and several strains were obtained from culture collections (Table 1). Isolates were originally selected to represent a range of nutritional types of interest in plant biomass conversion, and included 14 brown rot species, 22 white rot species, and 3 species that have an unknown decay type. All isolates are publically available through the Forest Mycology Culture Collection (University of Minnesota). Strain identity was determined or verified by DNA amplification and ITS sequencing (ITS1 and ITS4), matching with Genbank BLASTn database as previously described (Arenz et al., 2006). Isolates were maintained on 2% (w/v) potato dextrose agar (PDA) plates.

**TABLE 1:** Isolate information for fungi tested in microcosms for this study, including % mass loss (±standard error) from aspen wood decomposed 2 or 4 weeks by each fungus in soil-block microcosms.

**Fungus *Genus species***	**Rot type**	**Source**	**UMN code**	**GenBank accession**	**2-wk mass loss (%)**	**4-wk mass loss (%)**
*Antrodia sp.*	Brown	United States	202A	KC514838	0.48 (0.06)	7.10 (0.97)
*Antrodia carbonica*	Brown	FPL	753 FPL	KC514806	0.42 (0.20)	1.04 (0.15)
*Laetiporus squalidus*	Brown	Chile	ChBrnRt1	KC514808	3.63 (1.38)	17.55(7.64)^b^
*Laetiporus squalidus*	Brown	Chile	Ten. #91	KC514814	3.04 (2.15)	12.33(2.95)^b^
*Laetiporus squalidus*	Brown	Chile	Ach. #46	KC514825	2.44 (0.40)	16.57(4.42)^b^
*Postia* sp.	Brown	United States	PC2-2	KC514831	0.06 (0.05)	0.13 (0.09)
*Fistulina hepatica*	Brown	United States	FP-103444-T	KC514826	0.12 (0.11)	0.24 (0.04)
*Fomitopsis cajanderi*	Brown	United States	33A	KC514811	0.56 (0.14)	0.50 (0.31)
*Fomitopsis cajanderi*	Brown	MN	TAB 83	KC514827	6.78(4.17)^b^	22.01(0.01)^b^
*Gloeophyllum sepiarium*	Brown	United States	206A	KC514817	6.11(0.06)^b^	15.61(6.05)^b^
*Neolentinus lepideus*	Brown	United States	751	KC514815	2.30 (1.64)	30.48(0.02)^b^
*Oligoporus balsaminus*	Brown	United States	212A	KC514830	0.43 (0.29)	7.28 (0.14)
*Phaeolus schweinitzii*	Brown	United States	209	KC514818	1.62 (0.30)	14.31(0.35)^b^
*Pyrofomes demidoffii*	Brown	AZ	PJ-1	KC514835	0.33 (0.00)	1.17 (0.01)
*Conferticium ravum*	White	MN	BY1	KC514809	1.88 (0.50)	4.60 (1.55)
*Ceriporiopsis subvermispora^a^*	White	United States	105725 FPL	KC514810	0.80 (0.53)	9.48(4.55)^b^
*Aurantiporus* sp.	White	United States	Tyro292	KC514840	1.56 (0.41)	5.62 (1.04)
*Dichomitus squalens^a^*	White	United States	4C	KC514837	2.08 (0.33)	21.32(3.85)^b^
*Ganoderma sessile*	White	MN	GL-MN1	KC514839	9.13(1.92)^b^	23.17(2.94)^b^
*Ganoderma sessile*	White	MN	HoneyL1	KC514812	8.97(0.24)^b^	21.29(0.83)^b^
*Ganoderma tsugae^a^*	White	WI	WI-7C	KC514828	1.34 (0.16)	10.64(3.35)^b^
*Hymenochaete corrugata*	White	MN	H-2 MN	KC514813	2.67 (1.15)	14.44(0.06)^b^
*Inonotus dryophilus^a^*	White	MN	ID1	KC589014	0.18 (0.05)	3.74 (0.46)
*Irpex lacteus*	White	MN	34A	KC514829	9.97(0.17)^b^	19.58(0.32)^b^
*Peniophorella praetermissa*	White	Chile	Ten. #74	KC514832	0.06 (0.11)	0.10 (0.08)
*Perenniporia subacida^a^*	White	United States	11A	KC514821	4.63(0.25)^b^	9.37(0.01)^b^
*Phellinus arctostaphyli*	White	AZ	PM-1	KC589015	0.38 (0.04)	2.48 (0.47)
*Phellinus pini^a^*	White	MN	TAB 19	KC514836	0.37 (0.01)	1.67 (0.38)
*Phlebia brevispora*	White	United States	64C	KC514833	0.93 (0.21)	8.00 (0.96)
*Phlebia chrysocreas*	White	Chile	604	KC514834	4.38(0.43)^b^	9.58(4.08)^b^
*Phlebia* sp.	White	Chile	Park #82	KC514819	−0.20(0.05)	5.00 (0.96)
*Phlebia tremellosa^a^*	White	FPL	PRL 2845	KC514820	0.64 (0.50)	8.17(5.14)^b^
*Baltazaria* sp.^a^	White	NH	B360	KC514822	−0.08(0.10)	6.68 (1.52)
*Stereum hirsutum*	White	Chile	Calem #67	KC514824	1.31 (0.17)	12.34(2.93)^b^
*Trametes betulina*	White	United States	611A	KC514816	0.83 (0.08)	3.52 (0.26)
*Kretzschmaria hedjaroudei*	White	WI	303B	KC514841	0.44 (0.06)	1.12 (0.28)
*Jaapia argillacea*	Unk	Antar	Di44-5	KC514904	0.49 (0.52)	1.79 (0.40)
*Sistotrema brinkmannii*	Unk	Chile	Quin. 25A	KC514823	0.24 (0.12)	0.39 (0.24)
*Sistotrema coronilla*	Unk	Can	WBR-1	KC514807	0.11 (0.05)	0.07 (0.06)

*^*a*^Known to selectively degrade lignin. ^*b*^Indicates significantly greater mass loss than corresponding control (Dunnett’s multiple comparison, family error rate = 0.05). Control mass loss (no fungus added) for aspen averaged 0.1%. FPL, USDA Forest Products Laboratory Collection (Madison, WI, United States). All others available in University of Minnesota Forest Mycology Collection (St. Paul, MN, United States). AZ, NH, MN, and WI, State designators, United States; Can, Canada; Antar, Antarctica.*

### Wood Chemical Characterization

Aspen (*Populus tremuloides*) densities pre- and post-decay were measured in solid wood using dry weights (g; 100°C for 48 h) per fresh/green volumes (cm^3^). Aspen was selected again with a focus on plant biomass conversion. Sound and decayed wood was chemically characterized as in Sluiter and Sluiter (2010), without protein analysis. Substrates were Wiley-milled to 40 mesh with a Wiley Mill, and characterization of resulting powder was replicated four times. The sum of fractions (carbohydrates + lignin + ash + extractives + uronic acids + acetyl) averaged 94.6%, nearing mass closure of 100% of constituents accounted.

Acid-insoluble (Klason) was measured gravimetrically using 72% sulfuric acid as solvent, and acid-soluble lignin was quantified by spectrometer at a wavelength of 240 nm and with a extinction coefficient of 30, as recommended by Sluiter et al. (2008). Carbohydrates measured included glucan, largely representative of cellulose, as well as the hemicellulosic structural carbohydrates (xylan, galactan, arabinan, and mannan). These analyses included free monosaccharides that were soluble in water pre- and post-decay by fungi, which were as high as 2.11% of the total wood mass in decayed samples. Monosaccharides were separated via HPLC using an Aminex HPX-87P analytical column (Bio-Rad) and two in-line guard columns: Micro-guard Carbo-P and Micro-guard De-ashing (Bio-Rad). Mobile phase was degassed HPLC-grade deionized water (Sigma Aldrich) at an operating flow rate of 0.4 mL min^–1^. Operating column temperature was at 85°C, injection volume was 20 μL, and refractive index was used for detection. Standard response calibration curves were developed with reagent grade glucose, arabinose (Sigma Aldrich), galactose, xylose, and mannose (Acros Organics).

### Aspen Decomposition by Fungi

The intent of this study was not to demonstrate mass loss potential, but to capture early decay stages to match soluble sugar patterns with alkali solubility and, by design, accessibility for cellulases (data not shown). Aspen as 19 mm^3^ cubes were exposed to one of the 39 tested fungi for 2 or 4 weeks following the soil block test (American Society for Testing and Materials [ASTM], 1994), using one block per jar and five replicates per treatment as suggested by power analysis. Controls were placed in sterile soil jars for 2 or 4 weeks. Equal parts by volume of peat moss, potting soil, and vermiculite were wetted with distilled water and thoroughly mixed, adding 250 g of this mixture, lightly packed in 473 mL glass jars. Birch feeder strips (40 mm × 10 mm × 2 mm) were water-soaked under vacuum for 10 min and placed in parallel on top of the soil in each jar before autoclave-sterilization (twice at 121°C, 16 psi; 1 h, each run). Two fungal plugs were sterilely placed on the ends of each feeder strip and allowed to grow for two weeks before substrate addition. Treatments were doubled for 2 and 4 week destructive harvesting. Initial and final oven-dried mass was recorded after drying at 100°C for 48 h.

### Rot Type Indicators

Lignin loss relative to density loss (L/D, using %s) has been shown, as in Worrall et al. (1997) and Schilling et al. (2015), to be a useful index of carbohydrate selectivity (lower L/D; brown and soft rot) vs. lignin selectivity (higher L/D; white rot) among wood-degrading fungi. Worrall et al. (1997) used a soil-block design with 20 mm × 10 mm × 5 mm blocks, and Schilling et al. (2015) used wafers exposed inside petri plates over agar, with L/D calculated by the same methods. These L/D distinctions are only reliable when limited to wood within wood/bole decay classes II and IIII using Sollins (1982) and following Harmon et al. (2008) – a rot type cannot be assigned in class I when there is little discernable rot. In our case, this reduced the number of isolates from each study used in a composite of 78 isolates from this study combined with Worrall et al. (1997) and Schilling et al. (2015). A common threshold is 0.8 to distinguish brown and soft rot (<0.8) from white rot (>0.8).

Wood dilute alkali solubility (DAS) increases more during brown rot than during white or soft rot due to a combination of both lignin modifications and carbohydrate depolymerization (discussed in Schilling et al., 2015). We measured DAS for wood powder in 0.2 M sodium hydroxide as described by Shortle et al. (2010). The alkali solubility of degraded woody biomass offers a quick method of determining likely decay type and was used as a metric of decay type for samples degraded by unknown species based on our previous work (Schilling et al., 2015). This analysis, similar to L/D, is best done in decay class II/III if used in isolation to identify rot type, and the threshold of mass losses for significant DAS distinctions between rot types is often higher than the class I/II transition (Schilling et al., 2015). Instead of using DAS in isolation for rot type distinctions, we used this analysis across the entire range of mass losses to root best fits and demonstrate overall patterns of solubility.

Based on preliminary data from monosaccharide contents in decayed wood, the soluble sugar fractions for glucan and xylan were also measured with the hypothesis that higher soluble monosaccharides would be present during brown rot than during white rot. This hypothesis is at odds with Jurgensen et al. (1989) from field sampling performed at six conifer forest locations, but is in line with laboratory results noted by Cowling (1961). Cowling found in lab trials that brown-rotted sweetgum wood tended to have a greater concentration of “available reducing substances” than white-rotted wood in its hot water extractives, which would include water-soluble saccharides.

### Statistics

Statistical significance of mass loss was determined by comparing week 2 and week 4 samples with corresponding controls using Dunnett’s multiple comparison test (α = 0.05). Significance of differences in the progression of chemical component losses between white rot and brown rot was determined by first selecting a model fit for the loss of the chemical component as a function of total mass loss for both groups. Total mass loss was used as the independent variable in lieu of exposure time to control for variability among species in growth rate. Comparisons between the residual sum of squares (SS) from each group and the SS resulting from analysis when all data were pooled were made using *F* tests as described by Motulsky and Ransnas (1987). For all components, a linear model fit was deemed sufficient, as additional terms were not significant.

For DAS, a modified Michaelis–Menten equation offered the best model fit. This equation is described as follows:

$$D\,A\,S=D\,A\,S_{s\,o\,u\,n\,d}+\frac{\theta_{1}\times m_{l\,o\,s\,s}}{\theta_{2}+m_{l\,o\,s\,s}}$$

Where *DAS*_*sound*_ is the DAS of the undecayed controls, *m*_*loss*_ is the mass loss of the sample, and θ_1_ and θ_2_ to vary, with θ_1_ representing the asymptotic maximum DAS and θ_2_ representing the mass loss at which the DAS is at half of this maximum asymptote. Both decay type groups were separately fit to this equation by minimizing the residual SS while allowing θ_1_ and θ_2_ to vary. The same *DAS*_*sound*_ constant was used for both groups. After establishing the best fit equation for each groups, the equality of the variance about the decay type specific designated model fit was determined using Levene’s test. Likewise, Levene’s test was used to compare decay types for variance differences among the fit residuals for chemical component and sugar yield analyses.

A generalized linear regression model was used to assess the significance of the role of decay type in the release of free sugar over the course of degradation. Xylose and glucose yields were tested as response variables. Mass loss, decay type, and their interaction term were factors included in the model.

## Results and Discussion

### Mass Loss

Overall, wood mass losses were in line with the relatively short incubation period and generally confined results to early decay stages, as planned. Of the 39 isolates tested in initial screening with aspen wood, 24 caused a mean mass loss in excess of 5% after 4 weeks (Table 1). Seven isolates (2 BR and 5 WR) caused statistically greater mass loss than controls (*P* < 0.05) after 2 weeks and 18 isolates (7 BR, 11 WR) caused significant mass loss after 4 weeks. Eleven isolates, including those of unclear or unknown decay type (*Jaapia argillacea* and the two *Sistotrema* species), produced mean mass losses less than 2% after 4 weeks. Lignin selectivity metrics, which require a more strict threshold (>9% per aspen; Schilling et al., 2015), restricted L/D further to 13 isolates from this study that could be composited with previous data to pool L/D for 78 isolates, total.

### Wood Chemistry and Patterns of Decay

Aspen chemical composition analyses approached 95%, nearing full mass closure. Lignin content (wt%) was 20.4% (±0.2), glucan 44.2% (±0.2), and hemicellulose 22.7% (±0.2; xylan 15.8%). Uronic acid content was 3.0% (±0.0) and acetyl 4.2% (±0.0). Extractives were 3.0% (±0.3) and ash 0.2% (±0.0). This composition is comparable to previously described values for hardwoods/angiosperms (Rowell, 1984; Saddler and Mackie, 1990; Wang et al., 2012). Typical of hardwoods, hemicelluloses were predominantly composed of xylan and a relatively small proportion of mannan (2.8%, ±0.2), unlike a softwood like pine. Uronic acids and acetyl groups were abundant, also unlike pine, and ash and extractives contents were lower than what would be expected in non-woody grasses.

**FIGURE 1:**
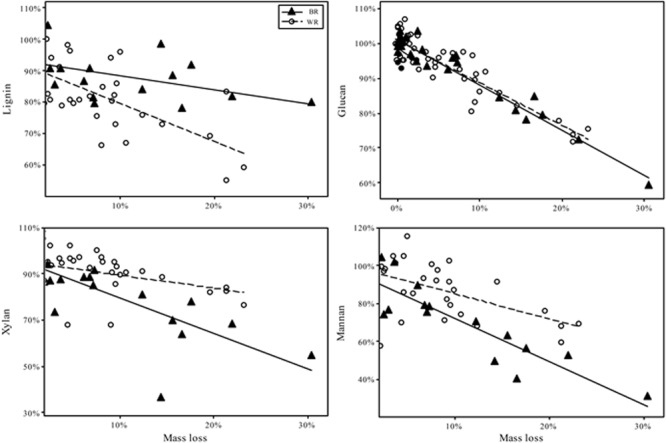
Percentage of chemical component remaining after fungal degradation to the indicated mass loss. Remaining percentage is based on the amount of component present in sound aspen. Trend fits are linear. Initial contents were, as % of dry mass (Standard error): Lignin 20.4% (0.2); Glucan 44.2% (1.1); Xylan 15.8% (0.2); Mannan 2.8% (0.2).

As test fungi decayed aspen, wood component loss patterns generally were characteristic of decay type for brown and white rot strains (Figure 1). White rot species removed lignin at a significantly faster rate than brown rot (11.8× faster) (*t*(65) = 4.44, *P* < 0.0001), and showed a collective pattern in line with simultaneous white rot. However, white rot species known to selectively degrade lignin (*n* = 8; noted in Table 1) caused lignin loss at a rate that was more than twice that of the other tested white rot fungi (Table 2A). These selective delignifying fungi also appeared to cause a slower rate of xylan loss than the other white rot fungi (*P* = 0.053), though these data were poorly fit by the model equation (*R*^2^ = 0.021).

**TABLE 2A:** Linear (*Y* = *M* × *X* + *B*) model fit coefficients, standard error (*S*), and the coefficient of determination (*R*^2^) for the% of component remaining in aspen wood as a function of mass loss after degradation by tested brown rot and white rot fungi.

	**Brown rot**				**White rot**				***T*-test**			**Levene’s test**	
***Component***	***M***	***B***	***S***	***R*^2^**	***M***	***B***	***S***	***R*^2^**	***T***	***P***	***DF***	***F***	***P***
Glucan	–1.335	1.009	0.023	0.970	–1.228	1.009	0.038	0.805	0.50	0.619	69	6.84	0.011
Xylan	–1.459	0.987	0.032	0.953	–0.938	1.016	0.030	0.813	2.65	0.010	58	0.24	0.625
Mannan	–2.253	0.965	0.061	0.922	–1.565	1.020	0.111	0.449	1.17	0.244	67	0.25	0.620
Lignin	–0.118	0.877	0.070	0.258	–1.395	0.945	0.090	0.497	–4.44	0.000	65	1.46	0.231

*Results of two sample *t*-tests comparing the effect of brown rot and white rot on the rate of loss and Levene’s test for equal variances about the model fits between the two decay types are also included.*

As often observed, brown rot hemicellulose losses tended to outpace white rot (Cowling, 1961; Kirk and Highley, 1973). Xylan and mannan loss rates in brown rot were 55 and 44% greater than white rot, respectively, but only the difference in the rate of xylan loss was statistically significant (Table 2B). Galactan was not present in an appreciable amount and arabinan loss was highly variable because of its minor contribution to overall mass of the wood. The rate of glucan loss was statistically identical in both decay types, but the variance about the linear fit for white rot was significantly greater than that for brown rot based on Levene’s test, with *P* = 0.0011 (Table 2B). Variance about model fits for other components also tended to be greater in white rot based on standard error, but the difference as compared with brown rot were not statistically significant.

**TABLE 3:** Linear (*Y* = *M* × *X* + *B*) model fit coefficients, standard error (*S*), and the coefficient of determination (*R*^2^) for the% of component remaining in aspen wood as a function of mass loss after degradation by known selective white rot species and all other tested white rot fungi.

	**Selective white rot**				**Other white rot**				***T*-test**		
***Component***	***M***	***B***	***S***	***R*^2^**	***M***	***B***	***S***	***R*^2^**	***T***	***P***	***DF***
Glucan	–1.116	0.994	0.038	0.77	–1.273	1.016	0.040	0.809	–0.76	0.457	23
Xylan	–0.115	0.906	0.051	0.021	–0.867	0.987	0.097	0.248	2.01	0.053	31
Mannan	–1.285	0.970	0.148	0.224	–1.334	0.992	0.135	0.286	–0.06	0.951	20
Lignin	–1.976	0.940	0.093	0.636	–0.930	0.906	0.084	0.333	2.13	0.046	20

*Additionally, the results of two sample *t*-tests comparing the effect of selective and other white rot species on rate of loss for each component are provided.*

### Fungal Rot Type Indicators

#### Dilute Alkali Solubility

Alkali solubility of brown-rotted wood typically increases in early decay relative to the DAS of sound wood, while white-rotted wood typically exhibits modest DAS increases (Cowling, 1961; Shortle et al., 2010). Schilling et al. (2015) showed for birch this threshold of distinction to be a DAS of 40%, a value that would vary by wood species and decay stage, but that held true for our isolates. Values for parameters θ_1_ and θ_2_ obtained by the fit of both decay types to a modified Michaelis-Menten equation were compared. Added to the initial DAS value (DAS of sound wood), θ_1_ represents the asymptotic maximum DAS, while θ_2_ represents the mass loss at which the DAS is at half of this maximum asymptote. Brown rot exhibited a significantly greater (*P* = 0.018) theoretical maximum DAS (θ_1_ = 0.54, vs. 0.06 for white rot).

**FIGURE 2:**
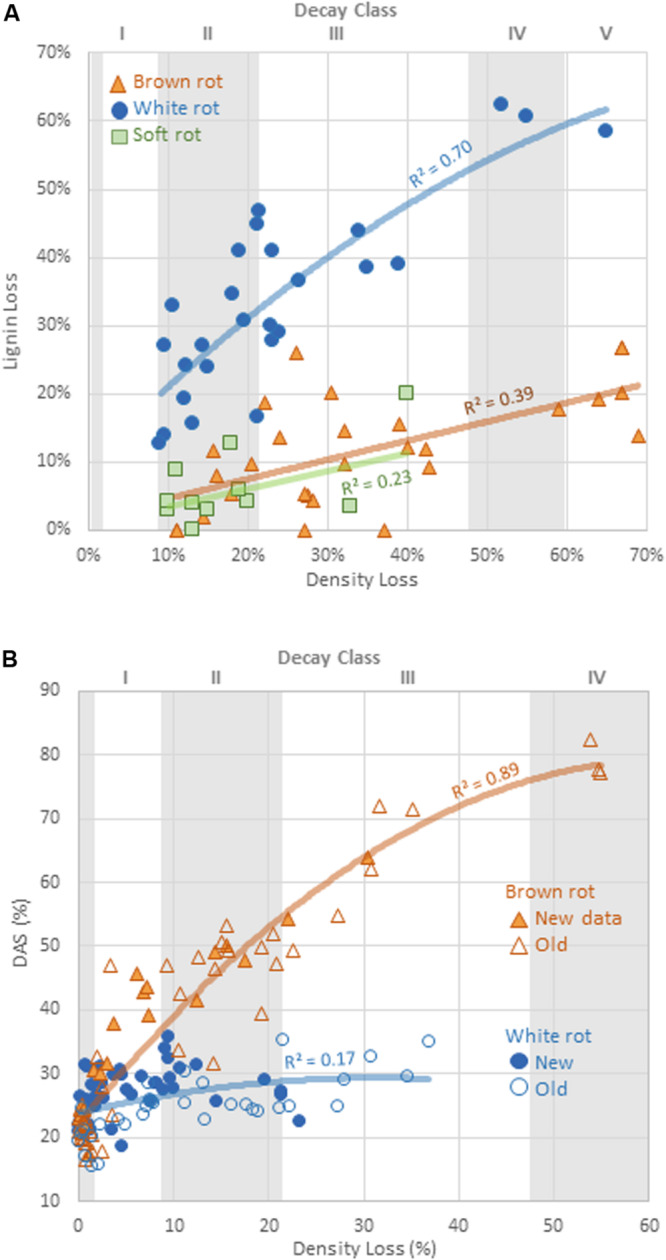
Comparison of **(A)** the rate of lignin loss and **(B)** change in dilute alkali solubility (DAS) relative to the rate of total mass loss for distinct decay types. Line fits are linear or exponential, showing best fits. Decay classes shown for wood substrates are specificto aspen, using Harmon et al. (2008) appendices.

Overall, the potential for DAS to distinguish brown from white rot was confirmed (Figure 2), and the patterns may be very useful for obtaining rot type information from isolates with poor BLAST query fits (<95% confidence) or that are complete unknowns. Using DAS, however, does bear caveats in the lab and in the field. First, we still lack data on soft rot alkali solubility effects, excepting a few targeted species tested over a half century ago (e.g., Savory and Pinion, 1958; Seifert, 1966; Henningsson, 1967). The screen by Worrall et al. (1997) is the only exception, giving the strongest indication that DAS is low for both soft and white rot fungi, but these studies have often been limited in scope by low wood mass losses. The second caveat, specific to field studies, is that early increases in DAS by brown rot fungi leave a signature in wood that cannot be erased in later stages, termed “legacy bias” in Schilling et al. (2015). These caveats, however, can be overcome by tracking lignin loss patterns as a complementary analysis, explained next.

#### Soluble Monosaccharides

**FIGURE 3:**
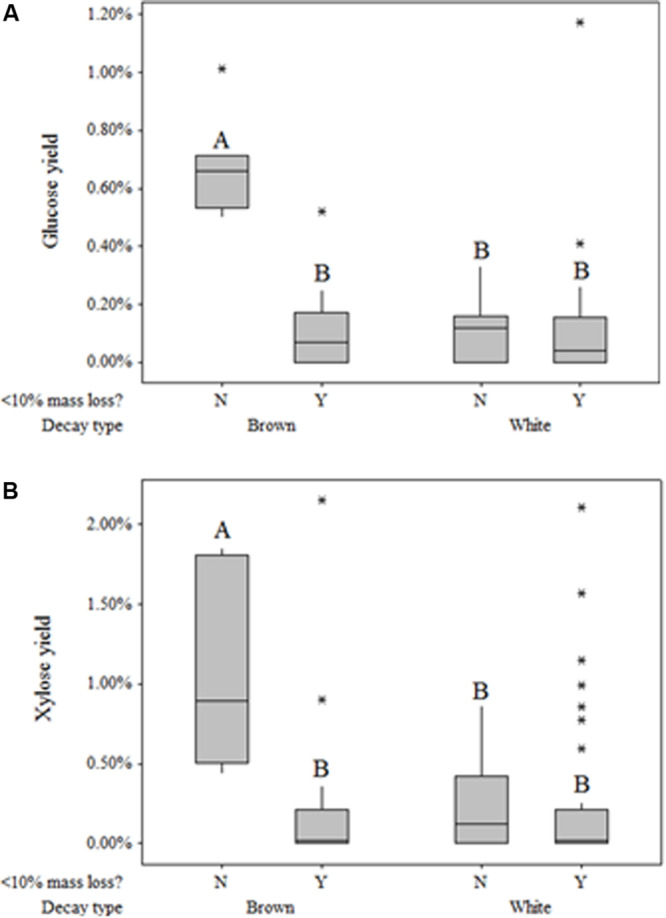
Box plots of glucose **(A)** and xylose **(B)** yield based on original glucan and xylan content, respectively, following pretreatment but preceding enzymatic hydrolysis, with respect to decay type and extent of decay (>10%). Plots within the same graph sharing the same letter are not significantly different based on Tukey’s *post hoc* comparisons.

Fungi degrading wood also solubilized monomeric sugars beyond those present in sound aspen at levels dependent on rot type (Table 3). Glucose and xylose yields resulting solely from fungal degradation were as high as 2.15 and 1.17%, respectively. General linear regression indicated that neither the main effect of decay type nor the interaction term between decay type and mass loss were significant factors in modeling xylose yield (*P* = 0.926, *P* = 0.053, respectively). For glucose yield, however, the interaction term between mass loss and decay type was highly significant (*P* < 0.001), though the main effect of decay type was not significant (*P* = 0.547). The significance of this interaction term was driven by the large difference in glucose yield between brown-rotted samples that exhibited more than 10% mass loss and those that did not exceed 10% mass loss. This mass loss distinction had no effect on either xylose or glucose yields of white rot (Figures 3A,B). As with glucose, xylose yield for brown rot was substantially higher than that of white rot when mass loss exceeded 10%.

**TABLE 3:** Soluble glucose and xylose after treatment with the indicated fungus for 2 or 4 weeks. Yields are expressed as a percentage of soluble sugar relative to the xylan or glucan content of untreated aspen.

**Species**	**Accession #**	**Decay type**	**Xylose (%)**		**Glucose (%)**	
			**2 weeks**	**4 weeks**	**2 weeks**	**4 weeks**
Control	–	–	0.06	0.09	0.09	0.03
*Aurantiporus sp.*	KC514840	W	0.11	2.11^a^	0.23	1.17^a^
*L. squalidus*	KC514808	B	0.19	1.85^a^	0.15	1.01^a^
*L. squalidus*	KC514825	B	0.10	0.52	0.10	0.71^a^
*P. schweinitzii*	KC514818	B	0.00	1.81^a^	0.00	0.69^a^
*N. lepideus*	KC514815	B	0.00	1.10	0.17	0.66
*F. cajanderi*	KC514827	B	0.18	0.50	0.13	0.59
*L. squalidus*	KC514814	B	0.10	0.89	0.12	0.53
*G. sepiarium*	KC514817	B	0.36	0.43	0.25	0.50
*G. sessile*	KC514812	W	0.08	0.42	0.21	0.33
*P. tremellosa*	KC514820	W	0.00	0.77	0.00	0.25
*Antrodia sp.*	KC514838	B	0.00	0.21	0.03	0.20
*J. argillacea*	KC514904	?	0.00	0.75	0.00	0.19
*D. squalens*	KC514837	W	0.05	0.22	0.15	0.16
*H. corrugata*	KC514813	W	0.00	0.12	0.10	0.15
*C. ravum*	KC514809	W	1.57	0.59	0.41	0.13
*I. lacteus*	KC514829	W	0.85	0.00	0.17	0.10
*P. chrysocreas*	KC514834	W	0.25	0.00	0.20	0.08
*Phlebia sp.*	KC514819	W	0.00	0.21	0.03	0.07
*O. balsaminus*	KC514830	B	0.01	0.22	0.01	0.07
*S. hirsutum*	KC514824	W	0.13	0.46	0.05	0.06
*S. coronilla*	KC514807	?	0.00	0.00	0.24	0.05
*P. subacida*	KC514821	W	0.00	0.20	0.00	0.04
*C. subvermispora*	KC514810	W	0.00	0.21	0.00	0.03
*Postia sp.*	KC514831	B?	2.15^a^	0.00	0.52	0.00
*P. arctostaphyli*	KC589015	W	1.15	0.00	0.26	0.00
*F. hepatica*	KC514826	B	0.90	0.00	0.23	0.00
*P. praetermissa*	KC514832	W	0.99	0.00	0.19	0.00
*I. dryophilus*	KC589014	W	0.02	0.00	0.16	0.00
*Baltazaria sp.*	KC514822	W	0.00	0.00	0.15	0.00
*P. pini*	KC514836	W	0.00	0.00	0.12	0.00
*T. betulina*	KC514816	W	0.00	0.00	0.11	0.00
*P. demidoffii*	KC514835	W	0.00	0.00	0.07	0.00
*K. hedjaroudei*	KC514841	W	0.01	0.00	0.04	0.00
*G. sessile*	KC514839	W	0.02	0.00	0.03	0.00
*G. tsugae*	KC514828	W	0.04	0.00	0.01	0.00
*P. brevispora*	KC514833	W	0.00	0.00	0.01	0.00
*A. carbonica*	KC514806	B	0.00	0.00	0.00	0.00
*F. cajanderi*	KC514811	B	0.00	0.00	0.00	0.00
*S. brinkmanii*	KC514823	?	0.00	0.00	0.00	0.00

*^*a*^Indicates significantly greater mass loss than corresponding control (Dunnett’s multiple comparison, family error rate = 0.05).*

This observation in “free” sugar patterns has rarely been identified, and results in the field have contradicted those in single-strain trials. Cowling (1961) found that brown-rotted sweetgum tended to have a greater concentration of “available reducing substances” than white-rotted sweetgum in all stages of decay when testing hot water extractives, which would include water-soluble saccharides. This observation of higher free monosaccharide in brown-rotted wood contradicts Jurgensen et al. (1989), who noted differences in soluble sugar concentrations for brown-rot and white-rot of native logs from six conifer forests. This distinction between lab and field may indicate a role for sugars released during brown rot in excess of the concentrations during white rot. Free sugar “cascade” effects on microbial communities have been observed in various other environments, including the microbiomes in the human digestive tract (Sonnenberg et al., 2010), soils (Luo et al., 2008; Chen et al., 2008), and forest litter (Scheu and Schaefer, 1998). These interactions have not been explored in wood decomposer communities, and may limit the ecological niche of brown rot fungi, given their diffuse ROS strategy for deconstructing wood.

#### Lignin Loss/Density Loss

**FIGURE 4:**
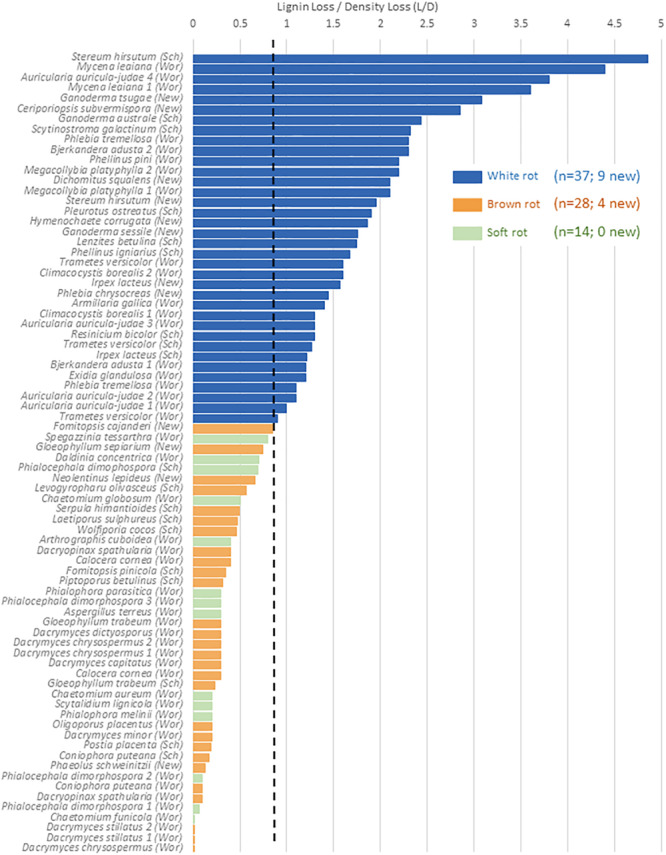
%Lignin loss relative to%Density loss (L/D) in aspen wood decayed by fungi with distinct nutritional modes, restricted to Class ll/lll decay. A O.S threshold (dashed line) distinguishing brown and white rot proposed by Worrall et al. (1997; “Wor”), and validated in Schilling et al. (2015; “Sch”), is complemented in this study (“New”). Gene count correlations with L/D are shown for the sum of genes from Table 2, including a separate correlation for PODs.

The relative selectivity of lignin removal from aspen was high for white rot fungi and low for brown rot fungi in this study, with soft rot patterns similar to brown rot (Figure 2). Data were combined with data from previous studies (*n* = 78, total) to demonstrate the binary distinction between white and brown rot (Figure 4), although it should be noted that *Daldinia* and *Hypoxylon* genera in the Ascomycota and designated as soft rot fungi have previously been shown to remove lignin more like white than brown rot fungi (Nilsson et al., 1989). White rot fungi that decayed aspen to Class II/III decay in our study were unanimously above the 0.8 threshold for L/D proposed by Worrall et al. (1997) and confirmed by Schilling et al. (2015). These new data build an even stronger case for using L/D to determine rot type outcomes in wood decay studies (Seifert, 1966; Schilling et al., 2015), but coupling with DAS for soft rot identification (Worrall et al., 1997). Although rot type is integrated into trait-focused DNA high-throughput sequencing tools such as FunGuild (Nguyen et al., 2016), the extrapolation of a functional wood decay outcome from operational taxonomic units (OTUs) remains limited by abundance quantification caveats and the assumption that the current snapshot of community can explain the history of rot. Measuring wood residues, directly, compiles the history of successional rot type dominance and offers a timely, useful complement to high-throughput sequencing.

The continuous nature of the L/D, however, offers an even bigger reward for linking molecular data to functional outcomes in two key ways: (1) by resolving the “gray” gradient of a rot type spectrum, and (2) by producing a continuous variable (from 0 to 5) with which to correlate other –omics data such as gene contents (Table 4). It essentially shifts data analyses from a bar graph with rot type as the independent variable on the *X* axis to enabling correlations along an L/D continuum. This gradient among the 78 isolate composite (Figure 4) shows the breadth of carbohydrate selectivity among wood degrading fungi that matches gene content in some, but not all cases. The continuous L/D also enables correlation of POD counts with lignin selectivity, also with a negative relationship (*r*^2^ = 0.23). These line fits are relatively poor and not shown here in Figures, but the lack of positive correlation is interesting, and the concept of linear regression with a direct measure of carbon release strategies should be very useful for future genomic studies.

**TABLE 4:** Gene contents, and corresponding lignin selectivity values (L/D), in annotated strains of brown and white rot fungal isolates used in this study.

**Fungus *Genus species*^∗^annotations noted, Table 1**	**Rot Type**	**GH5**	**GH6**	**GH7**	**Total GH**	**CBM_1**	**AA1_1 Lac**	**AA2 POD**	**AA3_2**	**AA5_1**	**AA9 LPMO**	**Total ALL**	**L/D**
*Coniophora puteana*	Brown	21	2	2	25	2	6	0	13	6	10	87	0.2
*Postia placenta*	Brown	17	0	0	17	0	2	1	24	3	2	66	0.2
*Gloeophyllum trabeum*	Brown	9	0	0	9	1	4	0	20	2	4	49	0.3
*Fomitopsis betulina*	Brown	18	0	0	18	0	2	1	17	4	3	63	0.3
*Fomitopsis pinicola*	Brown	19	0	0	19	0	5	1	16	4	4	68	0.3
*Calocera cornea*	Brown	23	0	0	23	1	0	0	13	3	0	63	0.4
*Laetiporus sulphureus*	Brown	20	0	2	22	0	7	1	26	5	2	85	0.5
*Serpula himantiodes*	Brown	25	1	0	26	6	5	0	14	5	5	87	0.5
*Wolfipora cocos*	Brown	18	0	0	18	0	3	1	8	4	2	54	0.5
*Leucogyrophana olivasceus*	Brown	26	1	1	28	8	6	0	9	5	12	96	0.6
*Neolentinus lepideus*	Brown	23	0	0	23	0	4	0	23	2	4	79	0.7
*Exidia glandulosa*	White	53	1	6	60	59	0	36	36	10	39	300	1.2
*Trametes versicolor*	White	22	1	4	27	23	7	26	17	9	18	154	1.3
*Armillaria gallica*	White	20	2	4	26	13	25	8	65	5	19	187	1.4
*Irpex lacteus*	White	26	1	4	31	26	0	10	21	7	16	142	1.4
*Bjerkandera adusta*	White	19	1	5	25	32	1	20	30	7	28	168	1.8
*Auricularia subglabra*	White	41	2	6	49	48	0	19	38	9	19	231	1.8
*Trametes betulina*	White	21	1	3	25	21	8	16	21	7	13	136	1.8
*Pleurotus ostreatus*	White	21	3	16	40	33	11	9	23	16	29	201	1.9
*Dichomitus squalens*	White	19	1	4	24	17	11	12	27	9	15	139	2.1
*Ceriporiopsis subvermispora*	White	18	0	3	21	16	9	17	17	3	9	113	2.9
*Stereum hirsutum*	White	20	1	3	24	17	15	6	40	8	16	150	3.5

*Gene counts accessed using JGI MycoBank.*

## Conclusion

The data generated in this study validate wood residue chemistry as a valuable tool for determining fungal rot type dominance and for linking omics information to a measurable outcome. Specifically, a continuous gradient of “gray rot” can be resolved by analysis of lignin selectivity (as L/D). This spectrum has a convenient division of brown (L/D < 0.8) from white rot (L/D > 0.8) that we reconfirmed here, and it can be used in tandem with a simple wood solubility test (as DAS) to help distinguish a third decay strategy, soft rot. These data offer a time-tested, but often overlooked quality control tool when attempting to extrapolate function from fungal gene contents, consequence from re-assembled secretomes in planta, or species dominance in field samples relying on relative OTU abundance estimates. Being able to tie genomic information and OTU tables to a consequence, measured in the wood, can greatly enable the promise of trait-based approaches trying to link the characteristics of fungi to their emergent roles.

## Data Availability Statement

The raw data supporting the conclusions of this article will be made available by the authors, without undue reservation.

## Author Contributions

JS, JK, BH, and RB designed the experiment. BH, RB, and RO collected, maintained and supplied isolates, with life history information, for lab trials. JS and JK performed the lab trials and analyses with the isolates. JK compiled information. JK and JS created the figures, tables, and manuscript, which was edited by all authors. All authors contributed to the article and approved the submitted version.

## Conflict of Interest

The authors declare that the research was conducted in the absence of any commercial or financial relationships that could be construed as a potential conflict of interest.
